# Improving properties of chitosan/polyvinyl alcohol films using cashew nut testa extract: potential applications in food packaging

**DOI:** 10.1098/rsos.241236

**Published:** 2024-12-04

**Authors:** Thuy Tien Dang, Lam Anh Thy Nguyen, Duc Tien Dau, Quy Sinh Nguyen, Thao Nhien Le, Thi Quynh Ngoc Nguyen

**Affiliations:** ^1^ Department of Food Technology, Ho Chi Minh City University of Technology (HCMUT), 268 Ly Thuong Kiet Street, District 10, Ho Chi Minh City 72506, Vietnam; ^2^ Vietnam National University Ho Chi Minh City (VNU-HCM), Linh Trung Ward, Thu Duc City, Ho Chi Minh City 71308, Vietnam

**Keywords:** chitosan, polyvinyl alcohol, extract, cashew nut testa, biodegradable, antioxidant

## Abstract

Cashew nut testa, a by-product of cashew nut processing, is abundant in phenolic compounds and exhibits strong antioxidant properties, making it a potential additive for enhancing the antioxidant properties of biodegradable films used in food packaging. This study explores the fabrication of biodegradable chitosan/polyvinyl alcohol films incorporating varying concentrations of cashew nut testa extract (CNTE; 0, 1, 2 and 3% v/v) and evaluates their physical, structural, mechanical, optical and antioxidant properties. The results demonstrate that increasing extract concentration generally increased the thickness, tensile strength, Young's modulus, thermal stability and antioxidant capacity of the films, while reducing the moisture content, swelling degree, elongation at break, and light transmittance. Specifically, the film with 3% extract showed approximately 11% lower moisture content and 31% lower swelling degree compared with the plain film. It also displayed the highest tensile strength and Young's modulus at 28.63 and 147.35 MPa, respectively. Microstructural analysis revealed that the incorporation of CNTE resulted in a smoother and slightly denser film structure. Antioxidant activity, determined by 2,2-diphenyl-1-picrylhydrazyl (DPPH) scavenging assay, was not detected in the plain film but increased with increasing extract concentration. The film with 3% CNTE exhibited the highest antioxidant activity of 58.93 µmol Trolox equivalents (TE) g^−1^ film. This study highlights the potential of CNTE as an effective edible additive for developing antioxidant and ultraviolet barrier films with improved mechanical strength and water resistance for food packaging applications.

## 1. Introduction

Food packaging plays a crucial role in preserving the quality, safety and shelf life of food products. Traditional food packaging materials, primarily derived from petrochemicals, have raised significant environmental concerns owing to their non-biodegradable nature and contribution to plastic waste. Additionally, the breakdown of plastic leads to the formation of microplastics, which are small plastic particles with size below 5 μm. Currently, these microplastics have widely spread across the oceans, rivers, soil and air [1], posing severe threats to both animal and human health [2]. The ingestion of microplastics by a diverse range of aquatic and terrestrial animals, including fish [3], birds [4], turtles [5], mammals [6,7] and crustaceans [8], has become an increasingly serious issue. These plastic particles take decades to hundreds of years to decompose [9]. Microplastics are now ubiquitous, and humans can unknowingly take them up through various pathways such as inhalation, ingestion and dermal contact [2]. These microplastic particles have the potential to reach different tissues and organs in the human body, including the brain, kidney and liver [2]. *In vivo* studies have shown that microplastics can induce some adverse health effects in animals, such as inflammation, immune system dysregulation and neurological dysfunction, implicating potential harmful effects of microplastics in human health. It is, therefore, crucial to urgently find alternative materials to replace or at least minimize the use of plastics.

Biodegradable films based on natural polymers such as chitosan, starch, cellulose and gelatin have emerged as a promising solution, offering both environmental benefits and functional properties. Among various biodegradable materials, chitosan has been gaining considerable interest owing to its innate antimicrobial activity, eco-friendly nature, low price because of its abundance, non-toxicity, biocompatibility and good film-forming ability [10,11]. The application of chitosan-based edible films in the food packaging industry has contributed to improving shelf life and sensory characteristics by leveraging the antimicrobial activity and good gas barrier property of chitosan, resulting in higher-quality products [11]. However, while chitosan-based films exhibit good potential, there remain challenges related to their mechanical strength, moisture resistance and antioxidant capabilities [12]. Films containing solely chitosan have inadequate mechanical properties and low water/water vapour resistance owing to the hydrophilic nature of chitosan [12,13]. Chitosan can be blended with other biodegradable polymers such as polyvinyl alcohol (PVA) to result in improved physical and mechanical composite films [14]. The biodegradability of chitosan/PVA composite film was proved previously [12]. Meanwhile, it is also desirable to enhance the antioxidant and ultraviolet (UV) shielding properties of food packaging materials to protect the food products from oxidation and photochemical damage caused by UV rays, which are among the primary factors contributing to food degradation [15]. In this regard, researchers have been looking for various plant extracts to enhance the functionality of chitosan-based films. The addition of different plant extracts was reported to enhance the antioxidant, antimicrobial and water-resistant properties of chitosan-based film [16–18]. The application of these films for the packaging of fresh meats, seafoods, nuts, oils and fruits resulted in improved quality and shelf life of the food products [13,16,19–21]. However, the incorporation of several plant extracts such as young apple extract [22] and *Cyclocarya paliurus* extract [23] was reported to reduce the tensile strength of the films. Therefore, identifying novel plant extracts that are able to enhance the antioxidant and mechanical strength of chitosan-based films is desirable to develop innovative packaging films.

Cashew (*Anacardium occidentale*) is a highly valuable nut crop predominantly cultivated in tropical countries, and its production is continuously growing. During cashew nut production, the cashew nut testa is often discarded as waste. Various studies have shown that cashew nut testa contains a significant amount of antioxidant compounds [24], which can be valorized to enhance the antioxidant properties of the chitosan-based films. Furthermore, the tannin compounds typically present in cashew nut testa [24,25] can act as cross-linking agents in the preparation of biodegradable films [24,26]. Therefore, cashew nut testa extract (CNTE) represents a promising material to enhance the properties of the biodegradable films, particularly in terms of antioxidant capacity, which is a desirable attribute for food packaging material [27]. In addition to the benefits of improving film properties, the valorization of cashew nut testa for biodegradable film production also contributes to increasing the economic value of this by-product while promoting environmentally friendly and sustainable practices. Utilizing CNTE in film production can simultaneously reduce waste and maximize the value extracted from the cashew production process. This approach aligns with the principles of sustainability and offers a promising solution for both the industry and the environment.

This study aims to investigate the effects of incorporating varying concentrations of CNTE (1, 2 and 3% v/v) on the physical properties, microstructural properties, mechanical properties, optical properties and antioxidant capacity of chitosan/PVA films. By assessing the film properties, we aim to elucidate the potential of CNTE as an effective additive in chitosan-based film. The findings from this research could pave the way for developing advanced biodegradable films with improved performance, addressing the dual challenges of food preservation and environmental sustainability.

## 2. Material and methods

### 2.1. Materials

Chitosan was purchased from Bio Basic, Canada. Polyvinyl alcohol was obtained from HiMedia, India. Unless otherwise stated, chemicals were obtained from Merck (Germany). Acetic acid and glycerol were obtained from China and met standards for analytical experiments. Cashew nut testa was supplied by a domestic agricultural producer in the local area. Absolute ethanol was obtained from Chemsol Vina, Vietnam.

### 2.2. Preparation and characterization of cashew nut testa extract

The extraction of cashew nut testa was conducted following the method described by Shiekh *et al.* [28]. Cashew nut testa with a moisture content of 10% was obtained from a cashew processing plant in Bien Hoa City, Vietnam. The cashew nut testa was ground into powder and then soaked in 70% ethanol at a ratio of 1 : 6 (w/v) for 12 h. The resulting mixture was subsequently filtered through Whatman filter paper to yield the crude extract. The crude extract was then subjected to rotary evaporation at 41°C to concentrate it by approximately 10 times, to result in the CNTE. The resulting concentrated extract was stored in a −80°C freezer until further use.

### 2.3. Evaluation of the properties of cashew nut testa extract

Total solid content was determined using a moisture analyser (MX-50; A&D Company, Japan) at 105°C.

To determine total phenolic content (TPC), 0.25 ml of CNTE was mixed with 3.75 ml of distilled water and 0.25 ml of Folin–Ciocâlteu reagent. The mixture was vortexed for 10 s. After incubating in darkness for 6 min, 0.75 ml of Na_2_CO_3_ 20% w/v was added to the mixture. The mixture was stirred in a vortex for 10 s and incubated in darkness for 60 min at room temperature. The absorbance of the mixture at 765 nm was measured in a spectrophotometer (V-730; JASCO, Japan). The TPC of the samples was presented as milligram gallic acid equivalent (GAE) per millilitre of CNTE (mg GAE ml^−1^).

The antioxidant activity of CNTE was determined using 2,2-diphenyl-1-picrylhydrazyl (DPPH) assay: 0.1 ml of the CNTE was mixed with 3.9 ml of methanol–distilled water (1 : 1) DPPH reagent solution (40 mg l^−1^). The absorbance at 517 nm was recorded using a spectrophotometer (V-730; JASCO) after the mixture had been incubated at room temperature for 30 min in darkness. The free radical scavenging ability was expressed as millimoles Trolox equivalents per millilitre of CNTE (mmol TE ml^−1^).

### 2.4. Preparation of cashew nut testa extract-incorporated chitosan/polyvinyl alcohol composite film

The CNTE-incorporated chitosan/PVA films were fabricated according to a previous report [18], with some modifications. First, chitosan (2 g) was dissolved in 50 ml of 2% (v/v) acetic acid aqueous solution and stirred at 60°C for 5 h. Meanwhile, PVA (0.75 g) was dissolved in 50 ml of distilled water and stirred at 60°C for 3 h. After that, the chitosan solution and PVA solution were mixed and stirred at 60°C for 1 h. Then, 0.6 g of glycerol was added and stirred at 50°C for 2 h. Next, different ratios of CNTE (0, 1, 2 and 3% CNTE (v/v), respectively) were added to the mixed solution and homogenized at 50°C for 30 min. Finally, the resulting mixture, at 0.3 g cm^−2^ density, was poured into a 7.5 cm diameter Petri dish and then dried at 45°C for 48 h. The resulting CNTE-incorporated chitosan/PVA films were peeled off at room temperature and stored in plastic Ziploc bags within desiccators before experimental use. The composite films incorporated with 0, 1, 2 and 3% CNTE were denoted CHI0, CHI1, CHI2 and CHI3, respectively.

### 2.5. Structural characterization of the films

The surface and cross-section microstructures of the films were visualized using a JSM-IT200 scanning electron microscope (SEM; JEOL, Japan). The film samples were coated with platinum. An acceleration voltage of 10 kV was selected to acquire the scanning electron micrographs. A magnification of 5000× was used to observe the surface while magnifications of 500× and 2000× were used to observe the cross-section.

### 2.6. Fourier transform infrared analysis

Fourier transform infrared (FT-IR) spectra of the films were recorded from 400 to 4000 cm^−1^ on a spectrometer (FT/IR-4700; JASCO) with a spectral resolution of 4 cm^−1^ and a total of 16 scans.

### 2.7. Evaluation of the physical properties of the films

#### 2.7.1. Colour properties

Colour parameters of the films, including L*, a*, b* values derived from CIE-Lab colour system were recorded on a colourimeter (Model CR-300; Konica Minolta, Japan). The total colour difference of film was determined using:


(2.1) $$\Delta E=\sqrt{{\left(L_{0}^{*}-\;L^{*}\right)}^{2}+{\left(a_{0}^{*}-\;a^{*}\right)}^{2}+{\left(b_{0}^{*}-\;b^{*}\right)}^{2}}\;,$$

where $L_{0}^{*}\;\left(98.96\right),a_{0}^{*}\left(-0.14\right)$ and $b_{0}^{*}$ (−0.38) are the colour values of a standard white plate, and $L^{*},a^{*}$ and $b^{*}$ are the colour values of the film.

#### 2.7.2. Film thickness

Thickness of the film samples was measured using a digital micrometer (Shahe, China) with an accuracy of 0.001 mm. Twelve random spots on each sample were selected to measure thickness for each treatment.

#### 2.7.3. Moisture content

The moisture content of the films was determined using a moisture analyser (MX-50; A&D Company) at a temperature of 105°C.

#### 2.7.4. Swelling

Swelling of the films was assessed following the procedure of Tagrida *et al.* [29]. Each film specimen (2 × 2 cm^2^) was dried at 105°C until a constant weight was obtained, which was recorded (*W*_1_). The dried film was then submersed in 50 ml of distilled water at room temperature for 1 h. After that, the sample was removed and placed on a Whatman filter paper for 5 min to eliminate the excess distilled water. The swollen sample was weighed (*W*_2_), and the swelling degree of the film was calculated using:


(2.2) $$Swelling\;degree\;\left(\%\right)=\frac{W_{2}-W_{1}}{W_{1}}\times 100,$$

where *W*_1_ and *W*_2_ (g) are the weight of the dried film specimen and the weight of the swollen specimen, respectively.

#### 2.7.5. Water solubility

To determine water solubility, film specimens (1 × 6 cm^2^) were first dried at 105°C for 24 h before recording their initial weight (*W*_1_). The dried films were then immersed in 30 ml of distilled water and shaken for 24 h at 120 r.p.m. at room temperature. After that, the film samples were removed and dried at 105°C for 24 h before their final weight (*W*_2_) was recorded. The water solubility (WS) of the film was calculated using:


(2.3) $$WS\;\left(\%\right)=\;\frac{W_{1}-W_{2}}{W_{1}}\times 100.$$

#### 2.7.6. Thermal stability

Thermal stability was evaluated using a thermogravimetric analyser (TGA55; TA Instruments Thermal Analyzers, USA). Thermogravimetric analysis (TGA) was carried out at a temperature ramp rate of 10°C min^−1^ from 30 to 600°C in nitrogen gas.

### 2.8. Evaluation of the mechanical properties of the films

Mechanical properties of the films, including tensile strength, elongation at break (EAB) and Young's modulus, were evaluated using a texture analyser (TA.XT Plus; Stable Micro Systems, UK). To conduct the measurement, the samples were prepared as 50 mm long and 20 mm wide specimens and securely held vertically between two miniature mechanical grips. The testing speed was set to 15 mm min^−1^. The initial distance between the two grip clamps was 30 mm. The tensile strength (MPa) was determined by dividing the maximum force (N) required to break down the film by the cross-section area (m^2^). EAB (%) was calculated by dividing the elongation of the film by the initial grip length and reported as a percentage. Young's modulus (MPa) was the slope of the linear segment of the stress–strain curve.

### 2.9. Light transmittance and opacity

A UV–visible spectrophotometer (V-730; JASCO) was used to evaluate the light transmittance in the wavelength range of 200–800 nm and opacity of the films. The films were cut into 30 × 10 mm specimens and placed into the UV–visible spectrophotometer, with air used as the reference. The opacity at 600 nm was calculated using equation (2.4) [30]:


(2.4) $$Opacity=\frac{-\log\left(T_{600nm}\right)}{a}\;\left(mm^{-1}\right),$$

where *T*_600nm_ is the fractional transmission at 600 nm, and *a* (mm) is the thickness of the film.

### 2.10. Evaluation of the antioxidant property of the films

The antioxidant activity of the composite films was evaluated using a method described in a previous study [31]. Film specimens of 0.2 g were cut into small pieces and mixed with 2 ml methanol. The mixtures were shaken mechanically (VS 15O; LAUDA, Germany) at 200 r.p.m. for 3 h. The supernatants were collected and used to determine the antioxidant activity using the DPPH free radical scavenging assay: 0.1 ml of the extraction of the CH–CNTE films with different CNTE ratios was mixed with 3.9 ml DPPH solution (0.5 mM), vortexed for 10 s and placed at room temperature in darkness for 30 min. The absorbance of the resulting mixture was recorded at 517 nm using a spectrophotometer (V-730; JASCO). The antioxidant activity of the CN–CNTE films was expressed as µmol Trolox equivalents per gram of film (µmol TE g^−1^ film).

### 2.11. Statistical analysis

All measurements were done in triplicate (*n* = 3). The average values and standard deviations for all tests were determined and then compared by using one-way analysis of variance (ANOVA) followed by Tukey’s multiple range tests. All analyses were conducted using Statgraphics Centurion 19. Differences in *p*-values of less than 0.05 were considered statistically significant.

## 3. Results and discussion

### 3.1. Antioxidant properties of cashew nut testa extract

The CNTE had a total solid content of 18.05 ± 0.42%. The extract exhibited a TPC of 102.51 ± 0.34 mg GAE ml^−1^ and an antioxidant activity of 1.11 ± 0.01 mmol TE ml^−1^. The TPC of the CNTE was much higher than those of other plant extracts employed in the preparation of antioxidant chitosan-based films, including Chinese chive root (3.21 mg GAE g^−1^) [21], *Nigella sativa* (1.39 mg GAE g^−1^), banana peel extract (3.48 mg GAE ml^−1^) [32] and *Ficus* leaf extract (2.1 mg GAE g^−1^) [33]. This comparison highlights the promising potential of CNTE for preparing chitosan-based films with high antioxidant activity.

### 3.2. Physical appearance and colour properties

**Figure 1.**
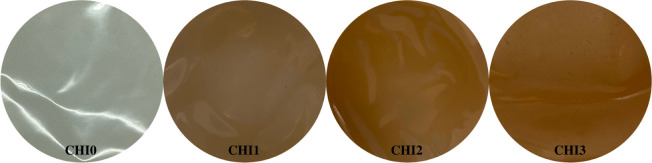
Physical appearance of CHI0, CHI1, CHI2 and CHI3 films.

The physical appearances of CHI0, CHI1, CHI2 and CHI3 film samples are presented in figure 1. The colour parameters (L*, a*, b*) of these film samples are summarized in table 1. The incorporation of CNTE resulted in darker films, as indicated by a gradual decrease of lightness values (L*) with increasing CNTE concentration from 0 to 3%. The darkening effect was commonly observed for the inclusion of plant extracts into chitosan-based films, which could be attributed to the light-absorbing properties of phenolic compounds in the extracts [16,18,34]. Furthermore, the addition of CNTE resulted in a shift toward a redder hue, as evidenced by higher values of green/red indicator (a*) in CHI1, CHI2 and CHI3 films compared with CHI0 film. The a* value gradually increased as the CNTE concentration increased from 1 to 3%, suggesting a shift toward a stronger reddish hue with increasing CNTE concentration. Conversely, the blue/yellow (b*) indicator decreased as the CNTE concentration increased from 0 to 3%. The total colour difference (Δ*E*) gradually increased as the CNTE concentration rose from 0 to 3%.

**Table 1.** Colour parameters, moisture content, swelling degree and water solubility of CHI0, CHI1, CHI2 and CHI3 films. Results are presented as mean ± standard deviation. Different superscript letters (a–d) within the same row indicate significant difference (Tukey’s comparison test, *p* < 0.05).

	film sample	CHI0	CHI1	CHI2	CHI3
color parameters	L*	36.43 ± 0.12^d^	28.16 ± 0.36^c^	27.49 ± 0.10ᵇ	26.72 ± 0.33ᵃ
	a*	2.63 ± 0.21ᵃ	6.09 ± 0.08ᵇ	6.39 ± 0.09ᵇᶜ	6.53 ± 0.14ᶜ
	b*	17.43 ± 0.07ᶜ	5.48 ± 0.45ᵇ	5.19 ± 0.27ᵇ	3.86 ± 0.13ᵃ
	∆*E*	65.07 ± 0.14ᵃ	71.31 ± 0.31ᵇ	71.99 ± 0.09^c^	72.67 ± 0.34^d^
moisture content (%)		13.68 ± 0.28ᵇ	13.55 ± 0.42ᵇ	12.44 ± 0.35ᵃ	12.17 ± 0.18ᵃ
swelling degree (%)		441.93 ± 7.52ᶜ	378.40 ± 2.42ᵇ	312.14 ± 9.82ᵃ	307.04 ± 10.29ᵃ
water solubility (%)		16.63 ± 0.94ᵃᵇ	15.32 ± 0.03ᵃ	17.66 ± 0.32ᵇᶜ	17.99 ± 0.25ᶜ

### 3.3. Fourier transform infrared analysis

**Figure 2.**
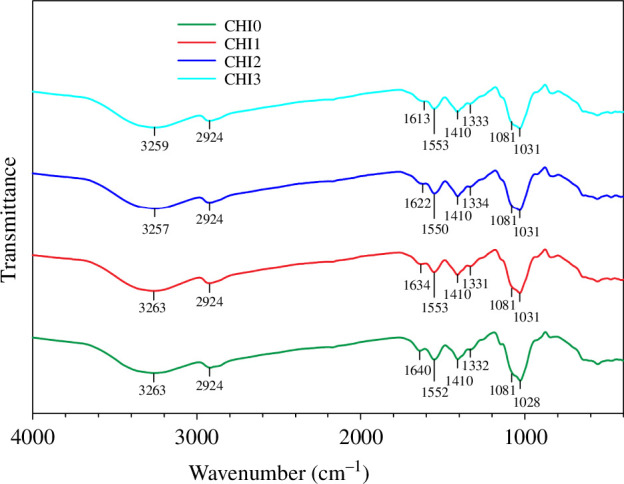
FT-IR spectra of CHI0, CHI1, CHI2 and CHI3 films.

As presented in figure 2, the CHI0 film exhibited a characteristic broad band at 3263 cm^−1^, which is attributed to the N–H and O–H stretching vibrations of chitosan and PVA molecules [16,35]. Additionally, CHI0 film exhibited the characteristic peaks at 2924 cm^−1^ for C–H stretching vibrations, 1640 cm^−1^ for amide I (C=O stretching vibrations), 1552 cm^−1^ for amide II (N–H bending vibrations), 1332 cm^−1^ for amide III (C–N stretching vibrations) and 1028 cm^−1^ for C–O skeletal stretching of chitosan [16,18]. The band at 1081 cm^−1^ corresponds to C–O stretching of PVA [35], while the band at 1410 cm^−1^ corresponds to CHI2 bending vibration of PVA and glycerol [16,35]. The addition of CNTE into the chitosan/PVA films caused a slight shift of the N–H and O–H stretching vibration band from 3263 cm^−1^ in CHI0 film to 3257 and 3259 cm^−1^ in CHI2 and CHI3 film, respectively (figure 2). Additionally, the amide I band also shifted from 1640 cm^−1^ in CHI0 to a lower wavenumber range (1634–1613 cm^−1^) in CHI1, CHI2 and CHI3. This observation is consistent with previous reports on the incorporation of natural extracts such as rambutan and persimmon peel, which also resulted in a shift of the amide I band to lower wavenumbers [16,34]. Moreover, the peak intensity of bands corresponding to amide I (1640–1613 cm^−1^), amide II (1552 cm^−1^) and C–O stretching (1028–1081 cm^−1^) was slightly lower in CHI1, CHI2 and CHI3 film compared with CHI0 film. These alterations in band position and intensity indicate the formation of hydrogen-bonding interactions between the phenolic compounds in CNTE and the functional groups of chitosan and PVA [16,34].

### 3.4. Film thickness

**Figure 3.**
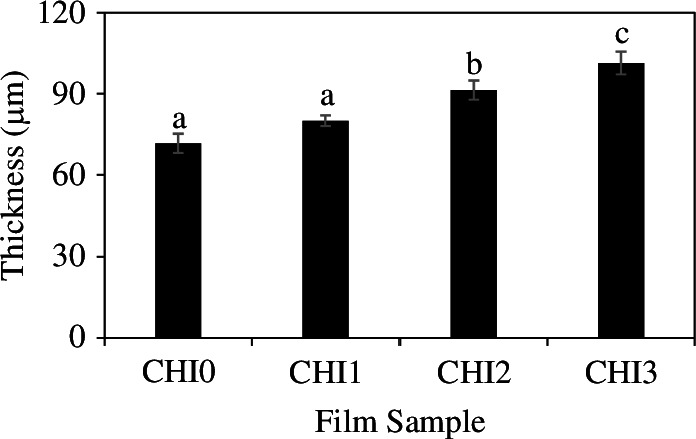
Thickness of CHI0, CHI1, CHI2 and CHI3 films. Different letters (a–c) indicate significant differences (Tukey’s comparison test, *p* < 0.05).

The thickness values of the composite films with different ratios of CNTE incorporation are presented in figure 3. Among the four composite films, CHI0 film exhibited the lowest thickness (71.67 ± 3.51 µm). The thickness of CHI1 film was not statistically significantly different from that of the CHI0 film. As the CNTE incorporation ratio increased from 1 to 3%, the thickness of the film significantly increased. The greatest thickness was recorded for CHI3 film (101.33 ± 4.16 µm), followed by CHI2 (91.33 ± 3.51 µm) and CHI1 (80.00 ± 2.00 µm). The increase in the film thickness can be attributed to the higher solid content and the intercalation of molecules in CNTE within the polymer matrix [16,23]. This observation is consistent with previous studies that have also reported an increase in the film thickness with the addition of various plant extracts, including *C. paliurus* extract [23], rambutan peel extract [16], thinned young apple extract [22], pistachio hull extract [36], walnut husk extract [37], hop extract [17] and neem extract [38], into chitosan-based films. These plant extracts typically increased the film thickness owing to the higher solid content incorporated into the film matrix. In contrast, a reduction in the film thickness was reported for the incorporation of rosary extract into chitosan film [39]. The thickness of packaging material is recognized as an important parameter in food packaging applications and has a significant impact on the food shelf life. Increasing the thickness enhances the gas/water vapour barrier properties by extending the path through which permeant molecules must travel. Moreover, increasing the film thickness can also reduce light transmittance and enhance the mechanical strength of the films [27]. Therefore, the increase in film thickness with the increasing level of CNTE incorporation is desirable. This result indicates that CNTE is a potential additive for enhancing the functionality of chitosan-based films as packaging materials.

### 3.5. Moisture content, swelling degree and water solubility

The incorporation of 1% CNTE did not significantly change the moisture content of the film compared with the plain film (CHI0). However, incorporating 2–3% CNTE into the film resulted in a lower moisture content compared with the plain film (table 1). This finding is consistent with previous studies that reported a reduction in moisture content of chitosan-based films with the addition of phenolic-rich plant extracts such as soybean seed coat extract [30], rambutan peel extract [16] and guava leaf extract [18]. CHI0 exhibited the highest moisture content owing to the abundant presence of hydrophilic NH_2_ and OH groups in the film, which are capable of forming hydrogen bonds with water. However, when CNTE was incorporated into the films, the phenolic compounds present in the extract interacted with these NH_2_ and OH groups of chitosan molecules, thereby preventing the interaction between the polymer molecules and water [34]. Furthermore, the hydrophobic nature of compounds such as catechin and quercetin, which are typically found in the CNTE extract [25], can also contribute to inhibiting the interaction of the film with water. Consequently, the moisture content was reduced in CNTE-incorporated films. The moisture contents of chitosan/PVA films incorporating 1–3% CNTE in this study were comparable to those reported for chitosan-based films incorporating 5–15% persimmon peel extract (16.34–12.72%) [34] but lower than those reported for chitosan films incorporating 1–3% rambutan extract (30.17–28.35%), 1–3% guava leaf extract (25.23–15.54%) [18], 0.5–1.5% hop extract (approx. 20–15%) [17] and 1–5% Chinese chive root extract (24.41–17.72%) [21].

The swelling degree of the film is an important parameter that indicates its water resistance property. As the concentration of CNTE increased from 0 to 3%, the swelling degree of CNTE-incorporated chitosan/PVA film significantly decreased from 441.93 ± 7.52 to 307.04 ± 10.29 (table 1). This reduction can be explained by enhanced cross-linking within the polymer matrix, promoted by the polyphenols in CNTE [18,40]. Especially, CNTE is typically rich in tannins, which were reported to act as cross-linking agents in the chitosan matrix [26,41]. Additionally, the hydrophobic nature of polyphenols in CNTE can also limit the swelling ability of the CNTE-incorporated films when exposed to water. The reduction in the swelling degree of CNTE-incorporated chitosan/PVA films indicates an increase in water resistance, which is desirable in food packaging applications. Similar findings regarding the reduction of the swelling degree were reported when plant extracts such as *Herba Lophatheri* extract [40], black chokeberry pomace extract [42], guava leaf extract [18] and *N. sativa* extract [13] were incorporated into chitosan-based film.

The water solubility of CHI1 and CHI2 was not statistically significantly different from that of CHI0, while CHI3 exhibited a slightly higher water solubility compared with CHI0 (table 1). As the CNTE concentration increased from 1 to 3%, the water solubility gradually increased. However, the difference in the water solubility of CHI3 and CHI2 film was not statistically significant. Similar observations were reported previously for the incorporation of *Sonneratia caseolaris* (L.) Engl. leaf extract and rosemary extract [43].

### 3.6. Thermogravimetric analysis

**Figure 4.**
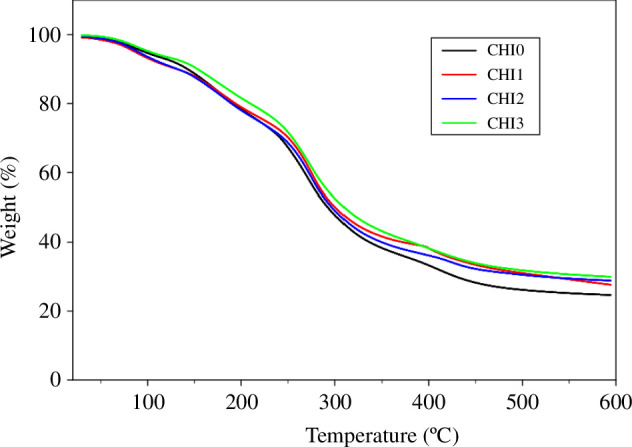
Thermogravimetric analysis of CHI0, CHI1, CHI2 and CHI3 films.

The effect of CNTE incorporation on the thermal stability of chitosan/PVA films was assessed using TGA (figure 4). Consistent with previous studies [18,30,44], all films displayed three primary stages of weight loss. The initial stage, occurring between 30 and 150°C, was attributed to the evaporation of water and residual acetic acid [18]. The second stage, from 150 to 250°C, corresponded to the decomposition of glycerol, while the third stage, from 250 to 600°C, was associated with the decomposition and depolymerization of polymer chains [18,30]. Notably, the CNTE-incorporated films (CHI1, CHI2 and CHI3 film) demonstrated lower weight losses compared with the plain film (CHI0). The total weight losses for CHI0, CHI1, CHI2 and CHI3 films were 74.84, 71.60, 70.58 and 69.84%, respectively. These findings suggest that the incorporation of CNTE enhanced the thermal stability of the films, with greater concentrations of CNTE correlating with increased thermal stability. A similar trend was observed for the incorporation of guava leaf extract [18], persimmon peel extract [34], black soybean seed coat extract [30] and pine park extract [44] into chitosan film.

### 3.7. Film microstructure

**Figure 5.**
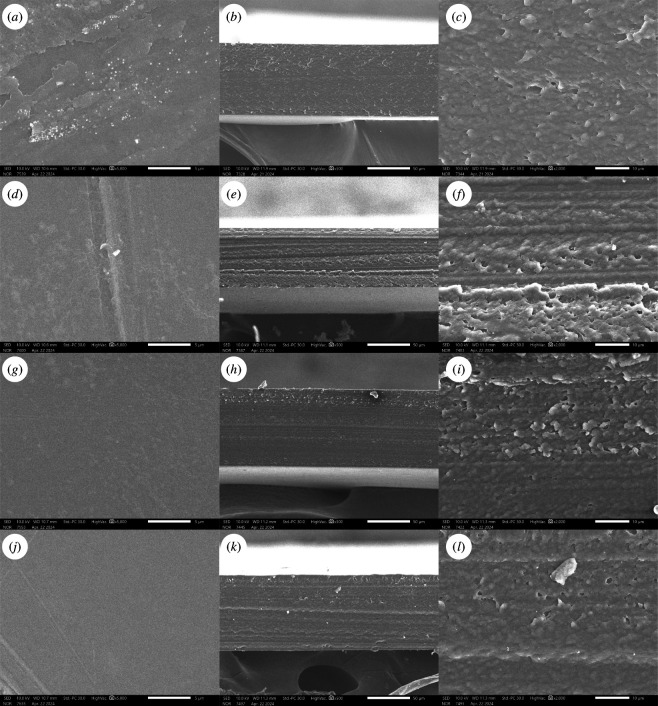
Scanning electron micrographs of the surface (left) and cross-section (middle and right) of CHI0 film (*a–c*), CHI1 film (*d–f*), CHI2 film (*g–i*) and CHI3 film (*j–l*). The magnifications of the micrographs are 5000× for the left image, 500× for the middle image and 2000× for the right image.

The microstructure of CNTE-incorporated chitosan/PVA films was exanimated using a SEM. The surface and cross-section of the films with different ratios of CNTE incorporation are shown in figure 5. Generally, as the CNTE addition ratio increased from 0 to 3%, the surfaces of the films appeared to be smoother. While the surface of the CHI0 film exhibited some extent of roughness, the CHI3 film displayed a homogeneous and smooth surface. Furthermore, the cross-section micrographs revealed a uniform structure with a slight improvement in the compactness as the CNTE addition ratio increased. These results indicate that the CNTE was uniformly distributed within the composite films and interacted effectively with the polymer molecules, resulting in a denser film structure. Similar results were reported for the addition of persimmon peel extract [34] and rambutan peel extract [16] into chitosan film, which resulted in a more continuous and uniform structure. The addition of *C. paliurus* extract [23] produced a rougher surface morphology but a more uniform cross-section structure. In contrast, the addition of many plant extracts such as apple peel extract [45], *N. sativa* L. extract [13], Chinese chive root extract [21], pine bark extract [44] and hop extract [17] was reported to result in heterogeneous film surfaces, microholes or less compact structures. The homogenous structure observed in CNTE-incorporated chitosan/PVA films in this study suggests that CNTE holds promising potential as an additive for enhancing the film structure in the fabrication of chitosan-based films.

### 3.8. Mechanical properties

**Figure 6.**
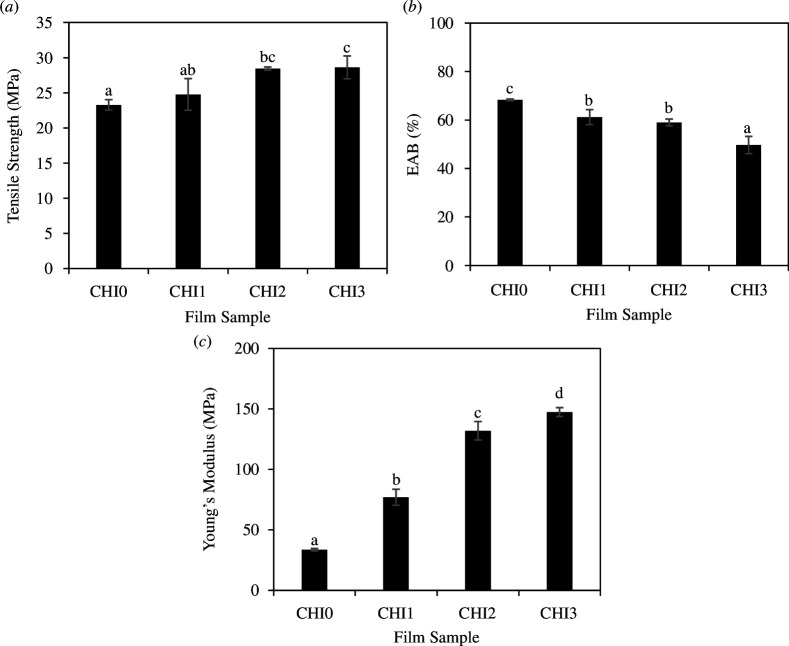
Mechanical properties of CHI0, CHI1, CHI2 and CHI3 films: (*a*) tensile strength, (*b*) EAB and (*c*) Young's modulus. Different lowercase letters (a–d) indicate significant differences in values within the same quantification (Tukey’s comparison test, *p* < 0.05).

The incorporation of CNTE had a significant impact on the mechanical properties of the chitosan/PVA films, as shown in figure 6. Tensile strength refers to the maximum stress that a film can withstand before breaking. Increasing the concentration of the extract from 0 to 3% led to a gradual increase in tensile strength from 23.28 ± 0.74 to 28.63 ± 1.63 MPa, with CHI3 exhibiting a tensile strength 23% higher than that of CHI0. Young's modulus gradually increased as the CNTE ratio increased. Young's modulus of the CHI3 film was approximately 4.4 times as much as that of the CHI0 film. The EAB represents the stretchability of the film. Corresponding to the increase in the tensile strength and Young's modulus, the EAB gradually decreased with increasing CNTE concentration from 1 to 3%. The EAB of the CHI3 film with 3% CNTE concentration was approximately 27% lower compared with the CHI0 film without CNTE. Despite the reduction in EAB at higher CNTE concentrations, the EAB of CHI3 with 3% CNTE was 49.66%, which is higher than the reported values for chitosan film incorporating black chokeberry pomace extract (EAB < 10%) [42], 2% guava leaf extract (EAB = 42.2%) [18], 10% grape seed extract (EAB = 21%) [46], 3% Chinese chive root extract (EAB < 40%) [21] and tannic acid (EAB < 30%) [26]. The EAB value of CHI3 film is comparable to the EAB of the chitosan film incorporating 5% rambutan peel extract (EAB = 51.73%) [16]. Different studies examining the influence of incorporating plant extracts on the mechanical properties of chitosan-based films have yielded heterogeneous results. The addition of black soybean seed coat extract [30] and rambutan peel extract [16] resulted in an increase in both tensile strength and EAB, while the incorporation of young apple extract [22], Chinese chive root extract [46] and banana peel extract [32] reduced both these parameters. Incorporating black chokeberry pomace extract [42] increased tensile strength but reduced EAB. Conversely, the addition of *C. paliurus* extract [23], *Moringa* extract [47] and hop extract [17] to chitosan-based film resulted in a reduction in tensile strength and an increase in EAB. The observed effects of CNTE incorporation on the mechanical properties of the chitosan/PVA films in this study could be attributed to the interactions between the phenolic compounds in CNTE and functional groups in the polymer chain, such as NH_2_ and OH groups (as indicated by the FT-IR analysis described above). These interactions led to the formation of additional cross-links between polymer chains, resulting in a strengthened cross-linked polymer network. Consequently, this led to a more rigid film structure with increased tensile strength and Young's modulus, but it reduced EAB. These observations are consistent with the microstructure of the CNTE-incorporated films (figure 5), which exhibited a slightly denser structure upon the addition of CNTE.

### 3.9. Light transmittance and opacity

Light transmittance and transparency are important properties of packaging materials for food products. Food oxidation is an undesirable process that reduces the shelf life of food. Exposure to light, especially UV light, can accelerate the food oxidation processes and hasten food deterioration [27]. Therefore, films with a light/UV barrier property are desirable for food packaging applications to prolong the shelf life of food [27]. This property helps mitigate the negative effects of UV rays on food [48]. Table 2 presents the light transmittance values of chitosan/PVA films incorporating various ratios of CNTE within the wavelength range of 200–800 nm. As the CNTE ratio increased from 0 to 3%, there was a gradual reduction in light transmittance at wavelengths 200–600 nm, except that the transmittance of CHI1 was higher than that of CHI0 film in the range of 350–425 nm (electronic supplementary material, figure S1). Specifically, the light transmittances in the UV region (200 and 280 nm wavelength) of the CHI1, CHI2 and CHI3 film were significantly lower than those of the CHI0 film. While the CHI0 film exhibited a peak of transmittance at 280 nm, transmittance at this wavelength was almost undetected for CHI1, CHI2 and CHI3 films (electronic supplementary material, figure S1; table 2). These observations indicate the superior UV and visible light barrier properties of the film incorporating 3% CNTE compared with the plain film. Similar findings were reported for the incorporation of various plant extracts, including rambutan peel extract [16], persimmon peel extract [34] and hop extract [17], into chitosan film. The addition of CNTE slightly increased the opacity of the composite films (table 2). Increase in the film opacity was generally observed with the incorporation of the plant extracts into the films [27].

**Table 2.** Light transmittance (%) and opacity of CHI0, CHI1, CHI2 and CHI3 films. Results are presented as mean ± standard deviation. Different superscript letters (a–d) within the same column indicate significant difference (Tukey’s comparison test, *p* < 0.05).

film sample	light transmittance (%) at different wavelengths (nm)								opacity (mm^−1^)
	200	280	350	450	500	600	700	800	
CHI0	0.02 ± 0.01ᵃ	2.27 ± 0.31ᵇ	4.11 ± 0.62ᵇ	43.37 ± 1.18ᵈ	55.80 ± 1.07ᵈ	67.83 ± 0.76ᵈ	73.81 ± 0.51ᵃ	77.36 ± 0.42ᵃ	2.35 ± 0.07ᵃ
CHI1	0.02 ± 0.02ᵃ	0.00 ± 0.00ᵃ	4.37 ± 0.31ᵇ	39.15 ± 0.42ᶜ	42.98 ± 0.45ᶜ	62.81 ± 0.19ᶜ	74.41 ± 0.04ᵃ	80.13 ± 0.07ᵇ	2.52 ± 0.02ᵇ
CHI2	0.01 ± 0.00ᵃ	0.01 ± 0.00ᵃ	0.67 ± 0.16ᵃ	30.19 ± 1.23ᵇ	34.19 ± 1.06ᵇ	60.72 ± 0.57ᵇ	75.51 ± 0.20ᵇ	82.07 ± 0.12ᶜ	2.58 ± 0.05ᵇ
CHI3	0.00 ± 0.00ᵃ	0.01 ± 0.01ᵃ	0.21 ± 0.10ᵃ	19.87 ± 0.09ᵃ	24.68 ± 0.07ᵃ	55.22 ± 0.01ᵃ	75.50 ± 0.07ᵇ	83.79 ± 0.13ᵈ	2.69 ± 0.00ᶜ

### 3.10. Antioxidant property

**Figure 7.**
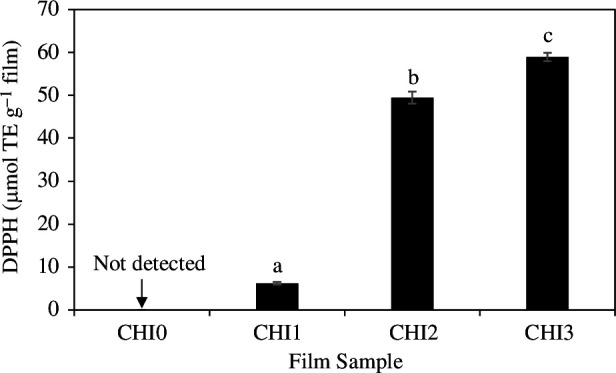
Antioxidant capacity of CHI0, CHI1, CHI2 and CHI3 films, as determined by 2,2-diphenyl-1-picrylhydrazyl (DPPH) assay. Different lowercase letters (a–c) indicate significant differences (Tukey’s comparison test, *p* < 0.05).

Cashew nut testa extract exhibits a strong antioxidant property as mentioned earlier. Therefore, the incorporation of CNTE was expected to enhance the antioxidant capability of the composite films. As shown in figure 7, the plain film, CHI0 did not exhibit detectable antioxidant activity. However, as expected, the incorporation of CNTE in CHI1, CHI2 and CHI3 film invested them with strong antioxidant activity. Furthermore, the antioxidant activity of the films increased significantly as the CNTE ratio increased from 1 to 3%. The antioxidant activity of the CHI3 film was 58.93 ± 0.95 µmol TE g^−1^ film, which was approximately nine times higher than that of CHI1 film (6.24 ± 0.39 µmol TE g^−1^ film). A similar increase in the antioxidant activity was commonly seen for the incorporation of plant extracts such as rambutan peel extract [16], persimmon peel extract [34], banana peel extract [32], rosemary extract [43] and mango leaf extract [20] into chitosan-based films. This increase was explained by the presence of phenolic compounds in the extract [21]. The CNTE was shown to exhibit high phenolic content, contributing to increased antioxidant activity of CNTE-incorporated film. The antioxidant property of the film is highly desirable in food packaging applications as it can mitigate the food oxidation process and enhance the quality and shelf life of food [27]. Chitosan-based films with enhanced antioxidant activity due to the incorporation of various plant extracts, including pine bark extract [44], Chinese chive root extract [21], walnut husk extract [37], cinnamon and tea extract [49], rosemary extract [43] and mango leaf extract [20], have been successfully utilized as packaging materials to reduce the oxidation and enhance the shelf life of food, including fresh meat, vegetable oil, fresh fruit, fresh fish and nuts. The strong antioxidant activity of the CNTE-incorporated films in this study suggests their promising potential for food packaging applications, particularly for foods with high fat content [16] and fresh fruits, where the antioxidant property of the film is desirable. These films can help preserve the freshness and quality of packaged food by preventing or slowing down oxidative reactions that lead to spoilage.

## 4. Conclusions

This study demonstrates the potential of cashew nut testa, a food by-product, as a valuable additive for enhancing the properties of biodegradable chitosan/PVA film for food packaging applications. The incorporation of CNTE into the chitosan/PVA films increased the film thickness, tensile strength, Young's modulus, thermal stability and antioxidant capacity, while reducing the moisture content, swelling degree, EAB and UV light transmittance. Furthermore, the incorporation of CNTE led to a smoother film surface and a denser film structure. The effects of CNTE on film properties were concentration-dependent, with greater effects observed at higher concentrations of CNTE. Overall, the CNTE-incorporated chitosan/PVA films show promising potential as antioxidant and UV light barrier packaging materials for food packaging applications for various food products. These films can effectively reduce the oxidation of oxidative-sensitive foods, including fresh meats, fresh fish, fresh fruits, nuts and vegetable/fish oils, when stored under refrigeration or ambient conditions, thereby enhancing product quality and shelf life. However, further research is needed to evaluate water and gas vapour permeability, antimicrobial activities, biodegradability and the overall food preservation efficiency of these films to fully assess their suitability for specific food packaging applications. The findings obtained from these studies can contribute valuable knowledge toward the development of sustainable and high-performance food packaging materials based on CNTE-incorporated chitosan/PVA films, paving the way for future innovations in biodegradable packaging solutions.

## Data Availability

Data are provided in Dryad [50]. Supplementary material is available online [51].
